# Screening of the siGPCR library in combination with cisplatin against lung cancers

**DOI:** 10.1038/s41598-022-21063-0

**Published:** 2022-10-17

**Authors:** Youngju Kim, Jieun Lee, Sumin Jeong, Woo-Young Kim, Euna Jeong, Sukjoon Yoon

**Affiliations:** 1 Department of Biological Sciences, Sookmyung Women’s University, Science building 404, Cheongpa-ro 47-gil 100, Yongsan-gu, Seoul, 04310 Republic of Korea; 2 College of Pharmacy, Sookmyung Women’s University, Seoul, 04310 Republic of Korea; 3 Research Institute of Women’s Health, Sookmyung Women’s University, Seoul, 04310 Republic of Korea

**Keywords:** Biological techniques, Cancer, Computational biology and bioinformatics, Drug discovery

## Abstract

The screening of siRNAs targeting 390 human G protein-coupled receptors (GPCRs) was multiplexed in combination with cisplatin against lung cancer cells. While the cell viability measure hardly captured the anticancer effect of siGPCRs, the direct cell count revealed the anticancer potential of diverse GPCRs (46 hits with > twofold growth inhibition, p-value < 0.01). In combined treatment with cisplatin, siRNAs against five genes (ADRA2A, F2RL3, NPSR1, NPY and TACR3) enhanced the anti-proliferation efficacy on cancer cells and reduced the self-recovery ability of surviving cells after the removal of the combined treatment. Further on-target validation confirmed that the knockdown of TACR3 expression exhibited anticancer efficacy under both single and combined treatment with cisplatin. Q-omics (http://qomics.io) analysis showed that high expression of TACR3 was unfavorable for patient survival, particularly with mutations in GPCR signaling pathways. The present screening data provide a useful resource for GPCR targets and biomarkers for improving the efficacy of cisplatin treatment.

## Introduction

G protein-coupled receptors (GPCRs) are major targets of therapeutic drugs against diverse diseases^1^. Their roles and functions have been recently reported in the cancer context, providing new strategies for cancer drug development. Several drugs or drug candidates targeting GPCRs are already in clinical use or in trials. For example, there are a few FDA-approved cancer drugs available, such as cabergoline, lanreotide, degarelix, plerixafor, vismodegib and raloxifene, that target DRD1 (dopamine receptor D1), SSTR (somatostatin receptor), GnRH (gonadotropin-releasing hormone receptor), CXCR4 (C-X-C chemokine receptor type 4), SMO (smoothened homolog) and ES (estrogen receptor)^2,3^. More compounds targeting GPCRs, such as CCR2/CCR4 (C–C chemokine receptor type 2/4), FZD4 (frizzled class receptor 4), and PE2/PE4 (prostaglandin receptors), are under clinical trials^4–6^. However, GPCRs have not been widely investigated as cancer targets, partly because of their low inhibitory outcome from conventional in vitro cytotoxicity (i.e., cell viability) assays^6^.

Cell viability measures based on mitochondrial activity have been widely used as surrogates for the mass change of cancer cells in screens. However, recent studies showed that cell viability measures from surviving cells also represent the resistant phenotype overcoming the effect of anticancer treatments; thus, the measure is not directly correlated with the cell number, particularly in the screen with the treatment of stress-inducing apoptotic anticancer agents^7–9^. In our previous study, we observed negative correlations between the total cell count and the viability of surviving single cells after treatment with anti-proliferative short interfering RNAs (siRNAs)^9^. However, the image-based direct cell count provided greater resolution than conventional viability measures for the quantification of the anticancer efficacy of siRNAs.

**Figure 1:**
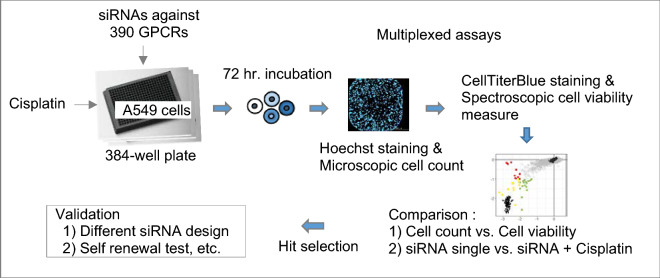
Overview of screening and data analysis procedure. Details are described in the “Methods” section.

Here, we carried out multiplexed screening of siGPCRs by using simultaneous measures of cell viability (mitochondrial activity) and image-based cell counts (Fig. 1). This multiplexing might effectively dissect the sensitive and resistant responses of cells against anticancer treatment, revealing further clues on the self-renewal ability of surviving cancer cells. Significant amounts of data have accumulated from forward screening campaigns using pooled short hairpin RNA (shRNA) or single-guide RNA (sgRNA) libraries against diverse cancer cell lines^10,11^. Data from well-based reverse screening using a siRNA library have limited availability from public domains but have advantages in compatibility with drug screen data and in employing diverse (or multiplexed) phenotypic assays^9,12,13^.

Cisplatin is a first-line chemotherapeutic against lung cancers and many other cancer types^7,8,14,15^. Although many combination targets for cisplatin have been reported, GPCRs have not been systematically investigated as potential targets for cisplatin combination therapy or for overcoming cisplatin resistance in cancers. Together with a single treatment of siGPCRs, we comparatively screened the combined treatment of siGPCRs with cisplatin against lung cancer cells. We expect that the present multiplexed screening provides sufficient resolution to capture the additive or synergistic effect of siGPCRs on cisplatin treatment.

## Results

### Data production from multiplexed screening of siGPCRs

A total of 390 siGPCRs were screened in both single and combined treatment with cisplatin against the A549 cell line (Supplementary Fig. S1). In this study, we carried out each of three screening repeats as a separate batch using independently cultured cells, thus improving the reproducibility of the outcome. The measure of the anticancer effect was multiplexed by adapting the cell viability assay together with simultaneous direct cell counting (see “Methods” for details). The z’-factor provides a quantitative measure of the data quality of the whole screening by measuring the resolution between positive and negative controls in the screening^16^ (Supplementary Fig. S2). Overall, the readout (Hoechst) of cell counting exhibited greater z’-factors than the cell viability measure (TiterBlue) in this screening. Most Z’ factors from the cell counting were above 0.5, while many Z’ factors from the cell viability assay did not reach 0.5. Consistent with the finding of our previous screening study^12^, this result shows that image-based cell counting provides greater resolution in the measure than the fluorescence-based cell viability assay, although the cell count measure has limited application to cells with minimal self-aggregation propensity in the course of proliferation. The complete list of screening results are available in Supplementary Table S1.

### Anticancer efficacy of siGPCRs in single and combined treatment with cisplatin

**Figure 2:**
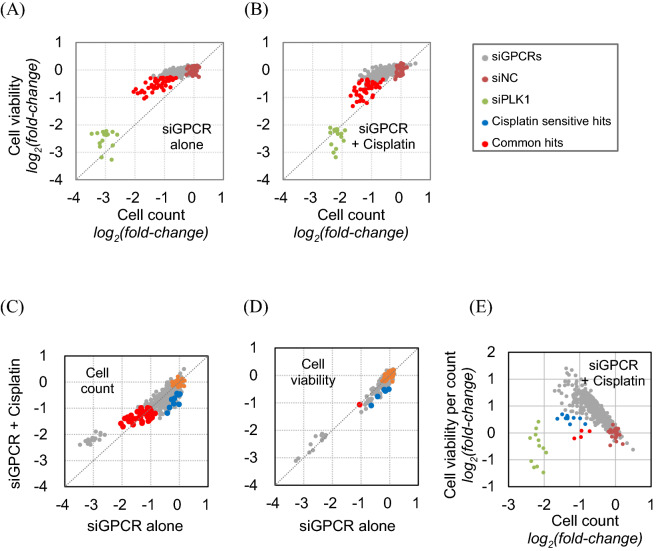
Distribution of anticancer effects of 390 siGPCRs with or without cisplatin treatment. Comparison of anticancer efficacy between cell count and cell viability measures in siGPCR treatment (**A**) and siGPCR + cisplatin combined treatment (**B**). Comparison of anticancer efficacy between siGPCR treatment and siGPCR + cisplatin combined treatment, measured by cell count (**C**) and cell viability (**D**). (**E**) Cell viability of surviving cells was plotted against the cell count for the siGPCR + cisplatin combined screening result.

The anticancer efficacy of 390 siGPCRs in the single treatment exhibited a wider distribution in the cell count assays than in the cell viability assay (Fig. 2A), although there was a general correlation in the results between the two assays. siGPCR hits with significant (p-value < 0.01) efficacy were found to have up to a fourfold (log2-fold) difference from siNC in the cell count assay, while the difference was up to twofold in the cell viability assay. The result of siGPCR screening in combination with cisplatin treatment also showed that the cell count provided a wider distribution of efficacy than the cell viability assay (Fig. 2B).

From the three repeats of screening, we identified a total of 46 siGPCRs as significant (p-value < 0.01) hits from cell count assays (blue and red dots in Fig. 2C). The complete list of hit genes is available in Table 1. Most of them showed prognostic RNA expression in LUAD patient samples (prognostic hit genes are colored with blue and red in Table 1). Their expression was significantly associated with patient survival in the Kaplan–Meier plot analysis. The common 37 siGPCR hits showed consistent cell growth inhibition (i.e., > twofold change) in both single and combined treatment with cisplatin (red dots in Fig. 2C). Furthermore, we identified nine siGPCRs exhibiting a significant (p-value < 0.01) increase in inhibition when combined with cisplatin (blue dots in Fig. 2C). However, most of the cell count hits were not recognized in the cell viability assay (Fig. 2D). Only a few hits were identified from the viability measure, and most siGPCRs showed nonsignificant outcomes.

**Table 1:**
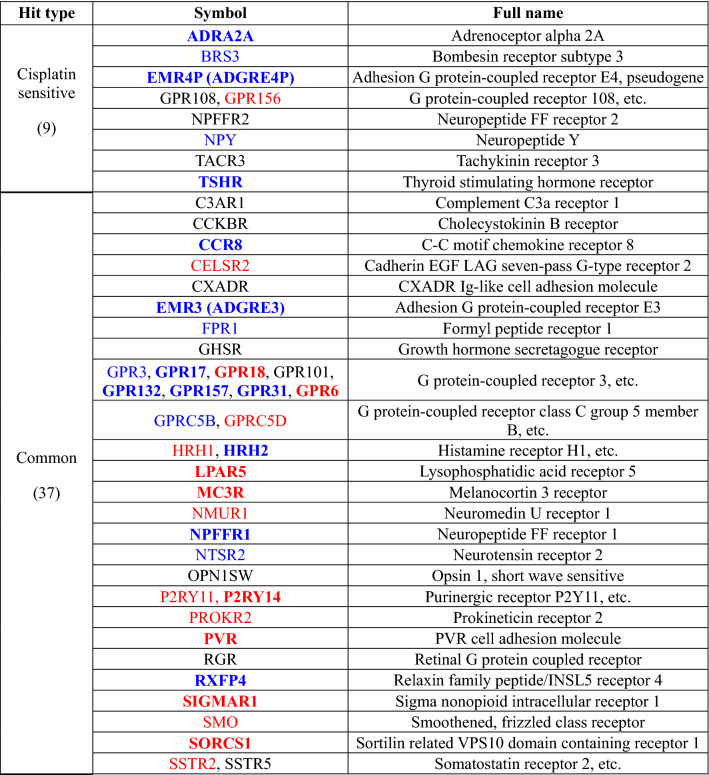
Summary of 46 siGPCR primary hits against A549 cells.

Blue/red colors represent favorable/unfavorable prognostic RNA expression of GPCR hit genes for patient survival. Regular and bold letters of blue and red colors represent p-value < 0.05 and p-value < 0.01 in the Kaplan–Meier plot, respectively. See details in the “Methods” section.

From the simultaneous multiplexed assays, we also measured the “viability per cell”, which was derived by dividing the well-based viability score by the cell count (i.e., the total number of cells in the well) (Fig. 2E). The results showed that the viability per cell generally increased as the cell count decreased. This implies that surviving cells from anti-proliferative treatments generally have higher mitochondrial activity (i.e., viability) than cells under normal conditions. siGPCR hits that significantly decreased the cell count exhibited a diverse range of viability per cell in surviving cells. Interestingly, the positive control, siPLK1 treatment, reduced the cell number without increasing the viability per cell in surviving cells.

### Validation of siGPCR hits for synergy with cisplatin treatment

**Figure 3:**
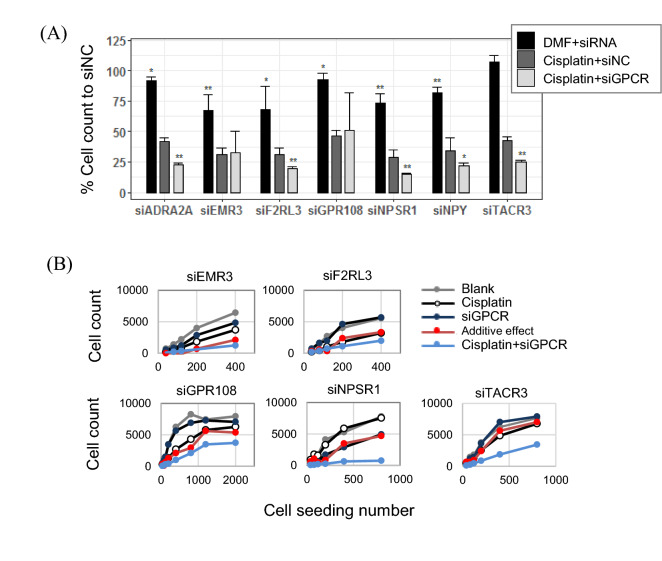
Validation of siGPCR hits in combination with cisplatin. (**A**) The anticancer efficacy of seven selected siGPCR hits was measured by the cell count in single and combined treatment with cisplatin. (**B**) Self-renewal analysis of A549 cells in fresh media after treatment with siGPCR and/or cisplatin. Additive effect is the sum of cell count changes in Cisplatin single treatment and siRNA single treatment. Detailed cell counts in maximum seeding numbers, are available in Supplementary Table S2. The treatment concentrations of siGPCRs were 10 nM for 4-probe pooling siGPCRs. Cisplatin treatment was 1 µM. */** represent p-value < 0.05/p-value < 0.01.

Among 46 siGPCR hits, we selected seven siGPCRs (siADRA2A, siEMR3, siF2RL3, siGPC108, siNPSR1, siNPY and siTACR3) for further validation (Fig. 3A). Their anticancer effects in single and combined treatment with cisplatin were measured by cell counting. Consistent with the primary screening result shown in Fig. 2C, they showed a marginal (but significant) anti-proliferation effect in the single treatment, except that siTACR3 failed to reproduce the significant effect. When combined with cisplatin, five of them, except siEMR3 and siGPR108, reproduced the significant enhancement in the anti-proliferation effect of the combined treatment in comparison to cisplatin treatment alone.

We also analyzed the self-renewal ability of surviving cells after siGPCR and/or cisplatin treatments (Fig. 3B). Surviving cells were transferred to fresh media, and their proliferation ability was measured by cell counting. We observed that all five siGPCRs (siEMR3, siF2RL3, siGPR108, siNPSR1 and siTACR3) markedly decreased the self-renewal ability of cancer cells when they were treated with cisplatin. Although they induced minimal change in the self-renewal ability by single treatment, they showed synergistic effects when combined with cisplatin (Supplementary Table S2 and Fig. 3B). The cell count decrease from the combination of siRNAs with Cisplatin was greater than the sum of decreased cell counts of single treatments of siRNA and Cisplatin (i.e., Red additive line in the Fig. 3B).

**Figure 4:**
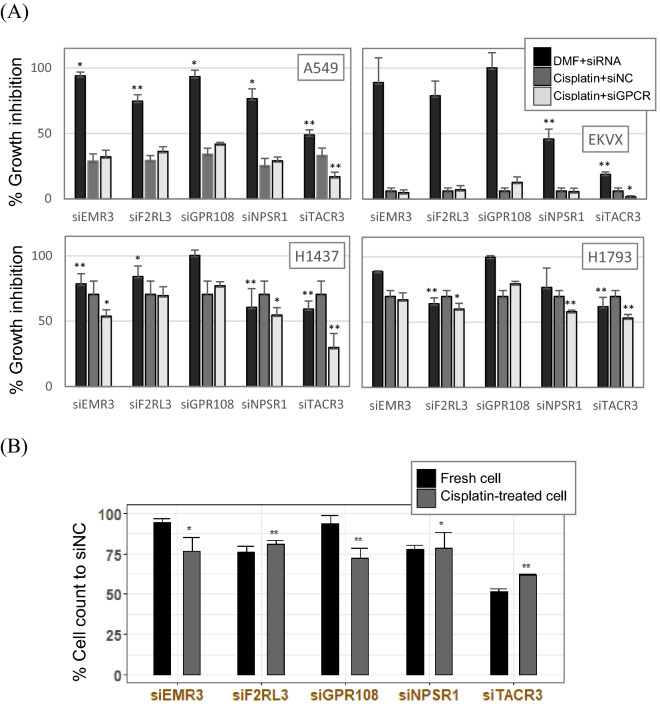
Validation of siGPCR hits in combination with cisplatin in diverse cell lines using advanced siRNA probes. (**A**) The anticancer effect of advanced siRNAs (20 probe pooling per gene) against hit GPCRs was tested in single and combined treatment with cisplatin against 4 different LUAD cell lines. (**B**) The anticancer effect of serial treatment with siGPCRs following cisplatin. The treatment concentrations of siGPCRs were 4 nM for 20-probe pooling siGPCRs. Cisplatin treatment was 1 µM. */** represent p-value < 0.05/p-value < 0.01.

To evaluate the off-target contribution to the anti-proliferation effect of siGPCRs, we carried out the knockdown experiment again with the increased number of diversified siRNA probe sequences per gene. For the primary screening, we used a pool of five siRNA probes per gene and treated them at a final concentration of 10 nM. Here, a total of 20 different probe sequences were pooled against a given target gene, and a 2.5-fold lower final concentration (4 nM) of the treatment was used for the second validation (Fig. 4). As a result, their marginal anti-proliferation effect in the single treatment was consistently reproduced (Fig. 4A). siTACR3 also showed significant efficacy. Synergistic inhibition with the cisplatin combination was only significantly observed with siTACR3 treatment. The combination of the other four siGPCRs with cisplatin showed similar efficacy to cisplatin single treatment. We also investigated the anti-proliferation effect of the serial treatment of cisplatin and newly designed siGPCRs (Fig. 4B). Surviving cells from pretreated cisplatin were compared with fresh cells under siGPCR treatment. All five siGPCRs showed greater anti-proliferation effects on cisplatin-pretreated cells than on fresh cells. In these secondary validations with newly designed siRNAs (Figs. 4A and 2D), siTACR3 exhibited a greater inhibitory effect than the other siGPCRs in both single and combined treatment with cisplatin.

### Prognostic RNA expression of TACR3 and associated mutations in patient samples

**Figure 5:**
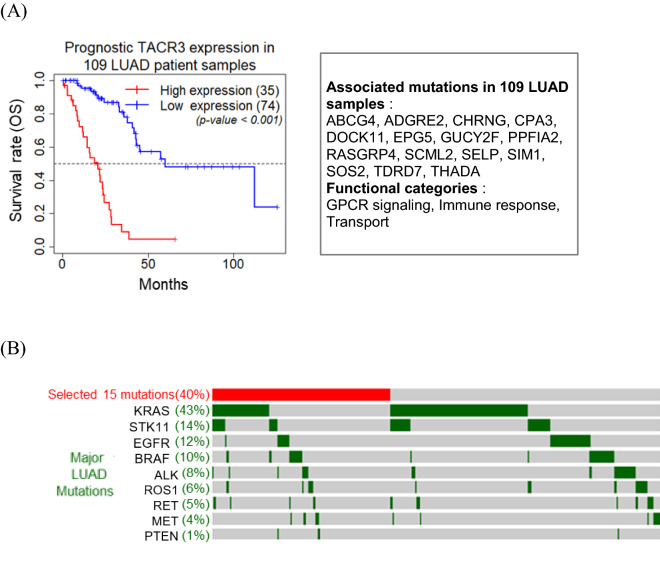
Association of TACR3 expression with patient survival in GPCR-mutant LUAD samples. (**A**) Kaplan–Meier plot of TACR3 RNA expression vs. overall patient survival. A total of 109 LUAD samples were selected based on the 15 mutations in the box on the right. (**B**) Oncoprint profile of 15 selected mutations and other major LUAD mutations. The total number of samples in this analysis is 268.

We investigated whether the RNA expression of TACR3 was associated with the survival rate of LUAD patients. In the extensive analysis of 490 patient samples derived from TCGA (https://portal.gdc.cancer.gov/) using Q-omics software (http://qomics.io)^17,18^, TACR3 expression was not associated with patient survival in any sample filtering conditions (OS/DFS, Median/Quartile, Stage-I/II/III/IV, Male/Female, Diverse subtypes, etc.) (Data not shown, see “Methods” for details). However, Q-omics software showed that overexpression of TACR3 was significantly associated with the survival rate in patient samples harboring one or more of 15 mutations (Supplementary Table S3). These mutations were found in GCPR signaling, immune response and transport functions. Under one or more mutations in these genes, TACR3 overexpression was unfavorable for LUAD patient survival (Fig. 5A). These mutations were found in 109 patient samples and were not specifically associated with other major oncogenic mutations (Fig. 5B). Together with the significant anti-proliferation effect of TACR3 knockdown (Fig. 3), the prognostic overexpression of TACR3 in patients harboring given mutations provides promising target-biomarker pairs for improved anticancer therapies.

## Discussion

The anti-proliferation potential of GPCRs has not been widely recognized in screening campaigns against cancers. Anticancer screening typically adapts cell viability assays measuring mitochondrial activity or ATP levels in live cells after treatment^19–21^. These viability measures are assumed to be correlated with the mass change of cancer cells in the screen, thus representing the anticancer efficacy of the treated agent. However, cell viability measures are not directly correlated with the change in cell count due to the increased mitochondrial activity in surviving cells after anticancer treatment^9^. Thus, when the image-based direct cell count was multiplexed with a cell viability assay, it provided improved resolution in identifying the anticancer potential of treatments^9^. The present screenings also showed that the cell viability measure provided poor resolution in quantifying the anticancer effect of 390 siGPCRs (Fig. 2). Over 10% of GPCRs exhibited significant anticancer potential from the direct cell count assay, while the cell viability measure showed only a few significant hits.

The viability per cell measure (Fig. 2E) might represent the resistance potential in surviving cells. A previous study showed that the viability per cell measure was correlated with the self-renewal ability of surviving cells after anticancer siRNA treatment^9^. We will further investigate whether this measure is correlated with the self-renewal ability of cells after the combined treatment of siGPCRs and cisplatin.

In the present screening, most of the primary hits were successfully reproduced in the validation steps (Fig. 3). We carried out three independent batches of screening using cells from different passages. The treatment concentration of siRNA was minimal at 10 nM and 4 nM for the Dharmacon and siPools siRNAs, respectively. We believe that the low concentration pooling of diverse siRNA probes (i.e., 20 probes per gene from siPools) effectively compromised the sensitivity and specificity against the target gene, thus minimizing the off-target effect.

Although TACR3 plays significant roles in physiological development and specifically in the human reproductive system, its role in carcinoma is unknown^22^. The present study showed that TACR3 might be a noble anticancer target, particularly with combined or serial treatment with cisplatin, one of the first-line drugs. The expression level of TACR3 was low in the normal epithelium and was highly elevated in tumor cells^22^. Interestingly, TACR3 overexpression was significantly associated with a poor survival rate when combined with mutations in other GPCR genes (Fig. 5). Although GPCR somatic mutations are widely present in LUAD samples, they have not yet provided therapeutic strategies. The present observation provides new insights into targeting GPCRs with GPCR mutations as patient stratification biomarkers.

## Methods

### Cell culture conditions

The non-small-cell lung cancer cell lines used in experiments included A549, EKVX (National Institutes of Health, National Cancer Institute, Frederick, MD, USA), H1793, and H1437(Korean Cell Line Bank, Korean Cell Line Research Foundation, Seoul, Korea). The cell lines were maintained in RPMI 1640 medium containing 10% fetal bovine serum (HyClone Laboratories, Logan, UT, USA) and 1% penicillin. For H1437, RPMI 1640, HEPES (GIBCO Laboratories, Grand Island, New York) was used. The cells were cultured in a humidified atmosphere of 5% CO_2_ and 95% air at 37 °C, and the culture medium was refreshed every two to three days.

### High-throughput siRNA screening

The siRNA screen was performed by using four pooled On-Target Plus siRNAs to target each of the 390 genes in the human GPCR siRNA library. Screening was performed in triplicate for four days in a 384-well plate format (400 cells per well). Each plate was supplied with a negative control siRNA (siNC; GE Dharmacon) and positive control siRNA (PLK1; GE Dharmacon) to distinguish sequence-specific silencing from nonspecific efficiency and the effects of knockdown. Reverse transfection was performed using a MultiFlo microplate dispenser (BioTek Instruments) with siRNAs (final concentration of 10 nM) and Lipofectamine RNAiMAX (Thermo Fisher Scientific, Waltham, MA, USA; 0.05 µl per well) diluted Opti-MEM (Thermo Fisher Scientific; 15 µl per well) in a black 384-well plate (Corning, Corning, NY, USA). After 24 h, each set was treated with 0.1% dimethylformamide (DMF) and cisplatin (final concentration of 1 µM). Cisplatin was obtained from Abmole Bioscience and dissolved in 100% DMF at a final concentration of 50 µM. Aliquots were stored at -20 °C and thawed and diluted (1 µM) immediately before use. After 72 h, the cells were assayed using the CellTiter-Blue Cell Viability Assay Kit (Promega, Madison, WI, USA) according to the manufacturer’s protocol, and the fluorescence produced was proportional to the number of viable cells. Plates were read on a SYNERGY H1 microplate reader (BioTek Instruments). After the cell viability assay, the cells were stained with Hoechst 33342 (Sigma–Aldrich, St. Louis, MO, USA), and the plates were imaged on a CYTATION3 imaging reader (BioTek Instruments) in 2 × 2 montage mode.

### Screening data analysis and hit selection

For the quality control metric in siRNA screening, Z’ factor is used which is defined as follows^16^:

$${\text{z}}^{^{\prime}} {\text{ - factor}} = {1}{-}{3}\left( {{\text{SD}}_{{{\text{siPLK1}}}} {-}{\text{SD}}_{{{\text{siNC}}}} } \right)/\left| {{\text{AVG}}_{{{\text{siPLK1}}}} {-}{\text{AVG}}_{{{\text{siNC}}}} } \right|,$$

where SD is for standard deviation and AVG is for average.

The range of z’-factor is negative infinity to one, with > 0.5 indicating an excellent assay and > 0 a borderline assay.

Raw data were converted into log2 scale to calculate the inhibitory effect of each siRNA: cell count and cell viability compared to the negative controls per plate. After normalization, negative values indicate that treating the target gene has a lower number of cells than the negative controls. All data were on a log2 scale, unless stated otherwise. Statistical significance was calculated by the independent 2-sample Student’s t test. Inhibitory siRNA hits were selected by combination of cisplatin and siGPCR transfection. The cisplatin-sensitive hits were selected with a p-value < 0.01 in combined treatment with cisplatin. The common hits were selected by p-value < 0.01 in both single and combined treatment with cisplatin. We calculated the cell counting value as a percentage for siNC-DMF. All data analysis including t test is done using Excel.

### Validation experiments of hit genes

Validation was performed to identify false positives. siRNAs from On-Target Plus siRNAs (siADRA2A, siEMR3, siF2RL3, siGPR108, siNPSR1, siNPY and siTACR3) were rescreened with A549 using the same transfection protocol of 10 nM by reverse transfection using Lipofectamine RNAi Max and Opti-MEM with DMF and a concentration of 1 µM cisplatin according to the manufacturer’s protocols. Repeated experiments were performed twice. Another validation experiment was conducted on five siRNAs (siEMR3, siF2RL3, siGPR108, siNPSR1 and siTACR3) from siTOOLs Biotech with A549, EKVX, H1437, H1793. siRNAs were transfected (final concentration of 4 nM) by reverse transfection using Lipofectamine RNAi Max and Opti-MEM and a concentration of 1 µM cisplatin with DMF. The experiment was repeated four times. For the anticancer effect, % Growth inhibition was defined as follows:

$$\% Growth inhibition= \frac{cell count\left(treatment, 96hrs\right)-cell count(0hr)}{cell count(control, 96hrs)-cell count(0hr)}\times 100$$

### Self-renewal experiment

A self-renewal experiment was conducted to identify how the pretreated chemical can affect the regeneration and growth of cancer cell lines even though it was replaced with fresh medium. We seeded the A549 cell line in a 96-well plate (Thermo Fisher) (2500 cells per well) for pretreatment. Transfection of siNC and siGPCRs was performed. After 24 h, the cells were treated with 1 µM cisplatin and DMF to identify four conditions (siNC-DMF, siNC-cisplatin, siGPCR-DMF and siGPCR-cisplatin). After a total of 96 h, the cells were harvested and seeded in 384-well plates by seeding number (40, 80, 120, 200, 400, 800, 1200 and 2000 cells per well) in fresh medium. After 96 h, the cells were assayed using Hoechst 33,342 (Sigma–Aldrich) to count the cells.

### siRNA transfection of cisplatin-pretreated A549 cells

For transfection of cisplatin-pretreated A549, we performed the same protocol with the A549 cell line prepared in advance. Cisplatin (1 µM) in the culture media was added to prepare cisplatin-pretreated A549 cells. We normalized the cell counting value as a percentage for siNC. The experiment was conducted with three repeats.

### Survival analysis

RNA sequencing data, mutation data and clinical data were obtained from The Cancer Genome Atlas (TCGA) GDC data portal^23^. The RNA sequencing data in FPKM (Fragments Per Kilobase Million) were converted to log2 (TPM + 1) (Transcripts Per Kilobase Million). Both FPKM and TPM indicate expression levels of RNA transcript. TPM was introduced as a measurement to correct the inconsistences among independent samples^24^. For calculation of survival analysis, the following patient details were obtained: vital status, gender, days to death, days to last follow-up and days to new tumor event after initial treatment from clinical data.

The genes were analyzed using the Kaplan–Meier method^25^ and the log-rank test^26^ via R. Gender (female, male and both) and stage (I, II, III and IV) were divided by TCGA clinical data. The patients were divided into high expression and low expression groups based on the median and quartile (high group > top 25%, low group < bottom 25%) gene expression among all patients for each gene. Overall survival (OS) was defined as the length of time from either the date of diagnosis or the start of treatment for a disease, such as cancer, to the date of death, and patients diagnosed with the disease who were still alive were censored. Disease-free survival (DFS) was defined from randomization to the first evidence of recurrence, second primary malignancy or death, whichever occurred first. To obtain a p-value indicating the significance of this analysis, the log-rank test^26^ was performed.

### Prognostic RNA expression

Survival analysis was calculated for 48 different measures using the TCGA dataset. The patients were divided according to the clinical data, including gender (female, male, and both) and stage (I, II, III, and IV). Gene expression groups were divided into medians or quartiles and calculated according to OS and DFS. Among the 48 survival p-values calculated in this way, the best p-value was used. We analyzed favorable and unfavorable prognostic gene expression^27^. Favorable prognostic gene expression was associated with a lower survival rate in the group with low gene expression, and unfavorable prognostic gene expression was associated with a lower survival rate in the group with high gene expression.

### Gene ontology database

We used GO (Gene Ontology) term to find out with which function each group of genes is associated. Among them, we focused on the biological process term. GO database were obtained from msigdb (https://www.gsea-msigdb.org/gsea/msigdb/).

### Oncoprint

Oncoprint was used to display mutation information for LUAD (Lung Adenocarcinoma) patients whose mutation information were obtained from TCGA. Data processing was done via R.

## Supplementary Information

Supplementary Information.

## Data Availability

All data generated or analyzed during this study are included in this published article and its supplementary information files.
