# Supplementary material for: Screening of the siGPCR library in combination with cisplatin against lung cancers
Source: Sci Rep. 2022 Oct 17;12:17358. doi: 10.1038/s41598-022-21063-0 (PMC9576725; doi:10.1038/s41598-022-21063-0)
Supplement: Supplementary file 1 — Supplementary Information. [file 41598_2022_21063_MOESM1_ESM.pdf]

Supplementary Materials for:

Screening of the siGPCR library in combination with cisplatin against lung cancers

Youngju Kim, Jieun Lee, Sumin Jeong, Woo-Young Kim, Euna Jeong, and Sukjoon Yoon

Supplementary Figure 1. Multiplexed screening of 390 siGPCRs in single treatment (A) and combined treatment with cisplatin (B) against the A549 lung cancer cell line. Gray circles represent individual siGPCRs. Black circles are the siNC. Blue circles are siPLK1 (positive control). Multiplexed readouts were obtained as the cell viability (RFU) and cell count (see Methods section for details). The treatment concentrations of siGPCRs and cisplatin were 10 nM and 1 μM, respectively. The unit of cell viability and cell counts is RFU (% of 0h) and number of cells (% of 0h), respectively.

(A) siGPCR single treatment.

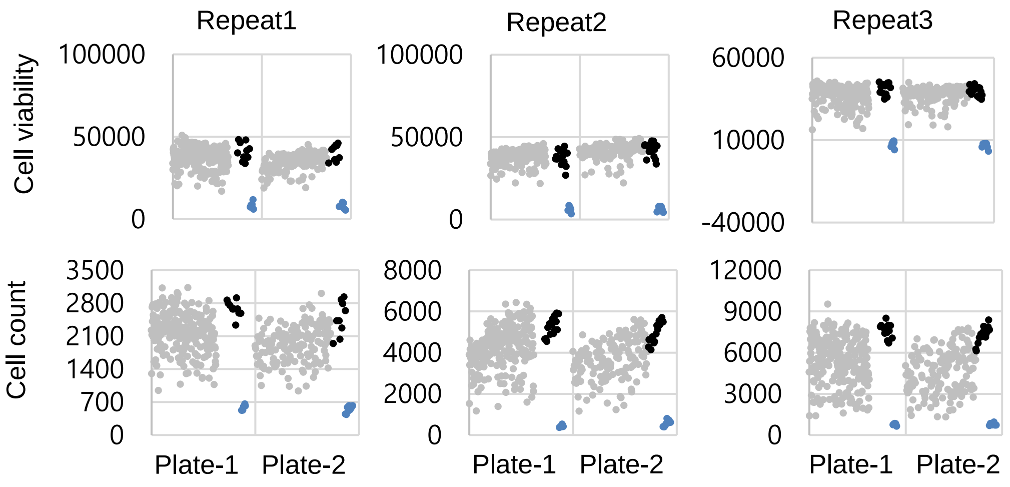

(B) siGPCR + cisplatin combined treatment.

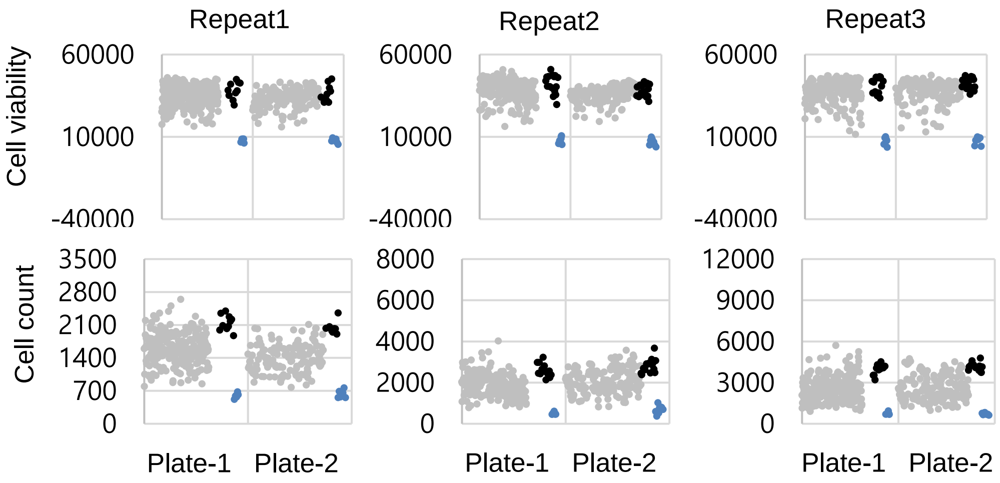

Supplementary Figure 2. Z' factor (data quality) of the screening results. The Z' factor was calculated for each plate to quantify the difference in readouts between siNC and siPLK1 treatments. Each dot represents a single repeat (see Methods for details of Z' factor).

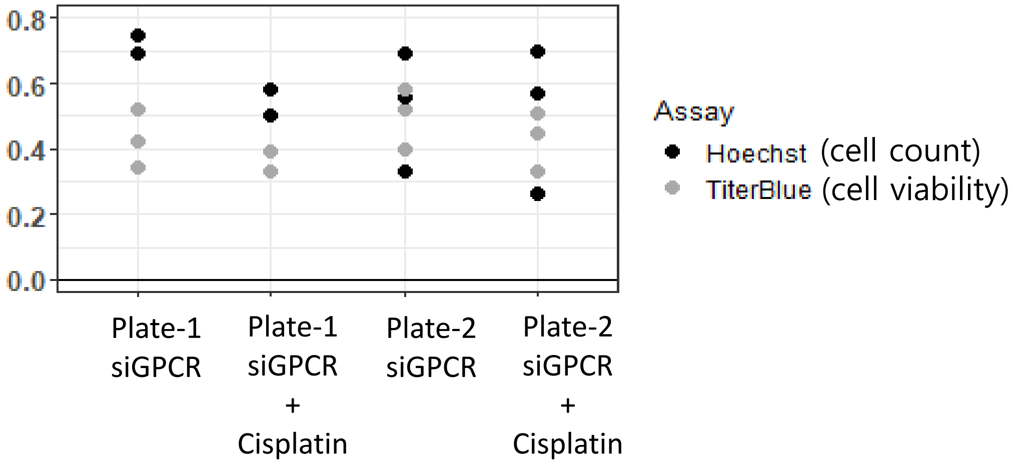

Supplementary Table 1. Screening results

Screening	TiterBlue		TiterBlue		Hoechst		Hoechst	
Treatment	DMF		Cisplatin		DMF		Cisplatin	
Gene symbol	AVG	p-value	AVG	p-value	AVG	pvalue	AVG	pvalue
EMR3	-0.642	0.000	-1.169	0.000	-1.083	0.000	-2.145	0.000
PVR	-1.044	0.000	-1.136	0.000	-1.684	0.000	-1.926	0.000
C3AR1	-0.855	0.000	-1.141	0.000	-1.905	0.000	-2.045	0.000
GPR3	-0.988	0.000	-1.321	0.000	-1.614	0.000	-2.402	0.000
SIGMAR1	-0.826	0.000	-1.210	0.000	-1.408	0.000	-1.926	0.000
P2RY14	-0.819	0.000	-1.095	0.000	-1.805	0.000	-1.995	0.000
OPN1SW	-0.702	0.000	-0.943	0.000	-1.969	0.000	-2.356	0.000
HRH1	-0.683	0.000	-0.988	0.000	-1.355	0.000	-2.126	0.000
PTGDR2	-0.747	0.000	-0.996	0.000	-1.026	0.000	-1.738	0.000
NPFFR1	-0.827	0.000	-0.997	0.000	-1.678	0.000	-2.113	0.000
LGR5	-0.617	0.000	-0.932	0.000	-1.435	0.000	-1.603	0.000
SORCS1	-0.658	0.000	-0.932	0.000	-1.542	0.000	-2.177	0.000
HRH2	-0.758	0.000	-0.906	0.000	-1.760	0.000	-2.275	0.000
GPR6	-0.655	0.000	-0.844	0.000	-1.249	0.000	-1.943	0.000
SSTR5	-0.427	0.000	-0.794	0.000	-1.302	0.000	-2.162	0.000
NMUR1	-0.416	0.000	-0.756	0.000	-1.017	0.000	-1.977	0.000
F2RL3	-0.400	0.000	-0.714	0.000	-0.847	0.000	-1.852	0.000
RGR	-0.800	0.000	-0.752	0.000	-2.042	0.000	-2.356	0.000
RXFP4	-0.549	0.000	-0.712	0.000	-1.430	0.000	-2.025	0.000
CHRM5	-0.320	0.002	-0.791	0.000	-0.671	0.000	-2.011	0.000
GHSR	-0.325	0.002	-0.722	0.000	-1.068	0.000	-2.054	0.000
PROKR2	-0.614	0.000	-0.671	0.000	-1.107	0.000	-1.761	0.000
CELSR2	-0.495	0.000	-0.669	0.000	-1.063	0.000	-1.857	0.000
FPR1	-0.597	0.000	-0.629	0.000	-1.337	0.000	-1.639	0.000
CHRM2	-0.370	0.001	-0.644	0.000	-0.717	0.000	-1.495	0.002
GPR101	-0.324	0.002	-0.643	0.000	-1.303	0.000	-2.341	0.000
HTR1E	-0.567	0.000	-0.635	0.000	-1.182	0.000	-1.618	0.000
MC3R	-0.417	0.000	-0.598	0.000	-1.315	0.000	-1.727	0.000
GPR18	-0.478	0.000	-0.545	0.000	-1.264	0.000	-1.940	0.000
GPRC5B	-0.310	0.001	-0.513	0.000	-1.074	0.000	-1.760	0.000
GPR31	-0.333	0.000	-0.518	0.000	-1.062	0.000	-1.757	0.000
SSTR3	-0.574	0.000	-0.540	0.000	-0.953	0.000	-1.450	0.000
FZD5	-0.635	0.000	-0.497	0.000	-0.794	0.000	-1.883	0.000
GPR50	-0.397	0.000	-0.509	0.000	-0.866	0.000	-1.726	0.000
MLNR	-0.423	0.000	-0.517	0.000	-0.949	0.000	-1.701	0.000
GPR157	-0.398	0.000	-0.508	0.000	-1.211	0.000	-1.924	0.000

OR1A1	-0.289	0.001	-0.482	0.000	-0.760	0.000	-1.535	0.000
LPAR5	-0.461	0.000	-0.490	0.001	-1.638	0.000	-2.192	0.000
P2RY11	-0.292	0.002	-0.463	0.001	-1.044	0.000	-2.052	0.000
BAI1	-0.298	0.001	-0.453	0.001	-0.606	0.000	-1.341	0.000
LPAR1	-0.390	0.000	-0.440	0.001	-1.272	0.000	-1.607	0.000
CCR8	-0.320	0.002	-0.423	0.002	-1.120	0.000	-1.875	0.000
TBXA2R	-0.295	0.004	-0.384	0.003	-0.910	0.000	-1.925	0.000
CXADR	-0.292	0.005	-0.376	0.003	-1.113	0.000	-2.098	0.000
GPR119	-0.268	0.003	-0.353	0.009	-0.803	0.000	-1.259	0.001
GPRC5D	-0.306	0.001	-0.352	0.009	-1.469	0.000	-1.787	0.000
SMO	-0.292	0.001	-0.338	0.013	-1.094	0.000	-1.702	0.000
S1PR4	-0.263	0.003	-0.345	0.014	-0.768	0.000	-1.370	0.000
GPR21	-0.256	0.004	-0.337	0.015	-0.918	0.000	-1.468	0.000
GPR83	-0.393	0.000	-0.313	0.015	-1.296	0.000	-1.736	0.000
SSTR2	-0.303	0.005	-0.275	0.020	-1.211	0.000	-1.981	0.000
TAAR1	-0.343	0.001	-0.262	0.027	-1.150	0.000	-1.507	0.002
GPR116	-0.225	0.009	-0.307	0.031	-0.837	0.000	-1.321	0.001
CCKBR	-0.306	0.001	-0.294	0.033	-1.033	0.000	-1.660	0.000
AGTR2	-0.234	0.007	-0.286	0.040	-0.989	0.000	-1.334	0.000
ADCYAP1R1	-0.242	0.005	-0.265	0.072	-0.843	0.000	-1.610	0.000
NPSR1	-0.362	0.005	-0.197	0.088	-0.950	0.000	-2.128	0.000
PROKR1	-0.293	0.004	-0.189	0.088	-0.782	0.000	-1.696	0.000
CCR3	-0.305	0.006	-0.112	0.259	-0.414	0.000	-1.138	0.133
MTNR1B	-0.274	0.011	-0.207	0.067	-0.547	0.000	-1.309	0.023
FZD2	-0.213	0.014	-0.484	0.001	-0.661	0.000	-1.467	0.000
CCR10	-0.210	0.015	-0.227	0.125	-0.843	0.000	-1.395	0.000
GPR64	-0.211	0.016	-0.171	0.330	-0.687	0.000	-1.198	0.004
ADORA3	-0.244	0.017	-0.362	0.004	-1.022	0.000	-1.733	0.000
FZD3	-0.204	0.018	-0.253	0.077	-0.927	0.000	-1.581	0.000
LTB4R	-0.200	0.020	-0.522	0.000	-0.860	0.000	-1.715	0.000
HTR1A	-0.208	0.021	-0.320	0.021	-0.643	0.000	-1.503	0.000
ADORA1	-0.226	0.024	-0.264	0.024	-0.640	0.000	-1.648	0.000
ADRA1D	-0.198	0.028	-0.228	0.137	-0.611	0.000	-1.254	0.001
VIPR1	-0.216	0.029	-0.370	0.005	-0.478	0.000	-1.333	0.019
GPR20	-0.185	0.029	-0.178	0.273	-1.043	0.000	-1.492	0.000
TAAR6	-0.222	0.029	-0.167	0.130	-0.404	0.000	-1.116	0.163
GPR17	-0.214	0.032	-0.304	0.011	-1.369	0.000	-2.082	0.000
CXCR6	-0.188	0.032	-0.267	0.058	-0.601	0.000	-1.196	0.005
CXCR7	-0.209	0.035	-0.199	0.075	-1.146	0.000	-1.635	0.001
TAAR9	-0.177	0.038	-0.208	0.173	-0.957	0.000	-1.752	0.000
GPR78	-0.179	0.038	-0.142	0.444	-0.682	0.000	-1.113	0.022

GPR111	-0.220	0.038	-0.042	0.562	-0.591	0.000	-1.067	0.243
GPR174	-0.216	0.038	-0.294	0.045	-0.406	0.000	-1.150	0.010
CNR2	-0.184	0.039	-0.333	0.014	-0.862	0.000	-1.283	0.001
GPR132	-0.212	0.040	-0.407	0.001	-1.017	0.000	-2.185	0.000
GLP1R	-0.212	0.041	-0.074	0.894	-0.191	0.041	-0.625	0.581
CCR4	-0.176	0.046	-0.356	0.008	-0.893	0.000	-1.157	0.010
CCRL2	-0.194	0.053	-0.443	0.001	-0.856	0.000	-1.742	0.000
TAAR5	-0.165	0.057	-0.067	0.937	-0.399	0.000	-0.698	0.923
FSHR	-0.163	0.059	-0.172	0.323	-0.393	0.000	-0.664	0.753
MC4R	-0.192	0.062	-0.390	0.002	-0.591	0.000	-1.670	0.000
TPRA1	-0.188	0.062	-0.508	0.000	-0.607	0.000	-1.753	0.000
TAAR8	-0.166	0.063	-0.357	0.008	-0.679	0.000	-1.408	0.000
S1PR5	-0.195	0.063	-0.211	0.063	-0.538	0.000	-1.610	0.001
DRD3	-0.183	0.065	-0.193	0.084	-0.771	0.000	-1.469	0.006
OPN1MW	-0.155	0.069	-0.173	0.291	-0.489	0.000	-1.303	0.001
FFAR1	-0.154	0.073	-0.165	0.337	-0.459	0.000	-1.277	0.001
GPR84	-0.153	0.078	-0.186	0.247	-0.499	0.000	-0.929	0.178
BDKRB1	-0.148	0.078	-0.642	0.000	-0.638	0.000	-1.834	0.000
GLP2R	-0.152	0.079	-0.141	0.454	-0.743	0.000	-1.554	0.000
DARC	-0.147	0.080	-0.256	0.083	-0.507	0.000	-1.111	0.016
ADRA1B	-0.147	0.081	-0.300	0.030	-0.708	0.000	-1.394	0.000
OPRL1	-0.172	0.081	-0.163	0.131	-1.062	0.000	-1.718	0.000
MC2R	-0.169	0.082	-0.279	0.019	-0.687	0.000	-1.589	0.001
GPR65	0.169	0.087	0.064	0.811	-0.210	0.009	-1.302	0.027
OXER1	-0.193	0.088	-0.261	0.030	-0.702	0.000	-1.728	0.000
MRGPRD	-0.143	0.091	-0.200	0.207	-0.628	0.000	-1.400	0.000
GPR182	-0.143	0.092	-0.382	0.005	-0.944	0.000	-1.733	0.000
GRPR	-0.179	0.097	-0.011	0.734	-0.136	0.035	-1.142	0.122
GRK1	-0.162	0.099	-0.221	0.053	-0.536	0.000	-1.484	0.003
OPN3	-0.138	0.104	-0.303	0.040	-0.453	0.000	-1.394	0.000
TAAR2	-0.138	0.106	-0.261	0.073	-0.877	0.000	-1.563	0.000
GRM7	-0.149	0.109	-0.036	0.838	-0.290	0.004	-1.329	0.001
ADRB2	-0.136	0.112	-0.142	0.443	-0.455	0.000	-0.908	0.233
PTGDR	-0.131	0.119	-0.307	0.027	-0.652	0.000	-1.157	0.010
MRGPRX3	-0.155	0.121	-0.340	0.006	-0.385	0.000	-1.782	0.000
CCBP2	-0.154	0.124	-0.100	0.302	-0.764	0.000	-1.304	0.023
GPR19	-0.148	0.127	-0.246	0.035	-0.732	0.000	-1.673	0.000
APLNR	-0.148	0.127	-0.273	0.021	-0.560	0.000	-1.510	0.002
LGR4	-0.157	0.129	0.033	0.998	-0.239	0.001	-1.120	0.154
GPR107	-0.128	0.136	-0.159	0.375	-0.319	0.001	-1.002	0.073
MC5R	-0.128	0.137	-0.187	0.249	-0.422	0.000	-1.168	0.007

CELSR3	-0.145	0.139	-0.338	0.007	-0.711	0.000	-1.861	0.000
MTNR1A	-0.123	0.139	-0.123	0.550	-0.753	0.000	-1.338	0.000
HTR3A	0.123	0.142	0.009	0.534	-0.074	0.440	-0.662	0.749
MRGPRF	-0.147	0.147	-0.205	0.069	-0.590	0.000	-1.540	0.001
EMR2	0.141	0.149	0.110	0.548	-0.066	0.341	-1.165	0.104
OPRM1	-0.123	0.151	-0.125	0.564	-0.277	0.003	-0.758	0.775
GRM2	-0.143	0.155	-0.461	0.001	-0.641	0.000	-1.944	0.000
GPR152	-0.144	0.163	-0.356	0.005	-0.369	0.000	-1.428	0.006
GPR4	-0.137	0.164	-0.216	0.058	-0.528	0.000	-1.403	0.008
HTR5A	0.132	0.174	0.082	0.705	-0.159	0.015	-1.733	0.000
F2R	-0.132	0.176	-0.190	0.086	-0.747	0.000	-1.702	0.000
SSTR4	-0.130	0.180	-0.361	0.003	-0.932	0.000	-1.888	0.000
GPR142	0.111	0.185	0.035	0.387	-0.153	0.102	-0.997	0.095
CHRM3	-0.111	0.187	-0.128	0.526	-0.552	0.000	-1.188	0.005
HTR2B	0.127	0.190	0.001	0.804	0.081	0.205	-1.153	0.144
LPAR4	0.126	0.191	0.161	0.318	-0.156	0.034	-1.164	0.099
GPER	-0.129	0.193	-0.152	0.153	-0.879	0.000	-1.602	0.001
HCRTR1	-0.129	0.194	-0.068	0.436	-0.290	0.000	-1.359	0.015
RRH	0.125	0.200	0.232	0.127	0.164	0.016	-0.275	0.007
CMKLR1	-0.107	0.201	-0.119	0.584	-0.373	0.000	-0.855	0.372
FKSG83	0.124	0.206	0.082	0.702	-0.184	0.011	-0.934	0.592
F2RL1	-0.123	0.208	-0.138	0.186	-0.379	0.000	-1.728	0.000
NPFFR2	-0.104	0.217	-0.504	0.001	-0.421	0.000	-1.837	0.000
RHO	-0.120	0.219	-0.184	0.113	-0.487	0.000	-1.355	0.013
CYSLTR2	0.118	0.220	-0.140	0.206	-0.211	0.002	-0.994	0.399
LPHN2	-0.118	0.223	-0.038	0.584	-0.472	0.000	-1.283	0.026
XPR1	-0.100	0.235	-0.114	0.613	-0.686	0.000	-1.381	0.000
NPR2	0.115	0.241	0.179	0.260	0.050	0.436	-0.934	0.591
TACR2	-0.098	0.244	-0.141	0.451	-0.427	0.000	-0.914	0.210
MCHR1	-0.098	0.246	-0.304	0.029	-0.725	0.000	-1.601	0.000
LPAR2	-0.109	0.258	-0.138	0.207	-0.329	0.000	-1.167	0.092
CCRL1	0.094	0.261	0.048	0.323	-0.052	0.573	-0.696	0.913
GPR98	0.108	0.269	0.077	0.732	-0.065	0.335	-1.147	0.132
CRHR1	-0.109	0.278	-0.107	0.294	-0.561	0.000	-1.563	0.001
SORCS2	-0.106	0.278	-0.230	0.048	-0.401	0.000	-1.241	0.044
LPAR6	-0.090	0.294	-0.208	0.225	-0.783	0.000	-1.220	0.002
GPR15	-0.103	0.296	-0.269	0.023	-0.492	0.000	-1.288	0.028
HRH4	-0.105	0.308	0.023	0.934	-0.379	0.000	-1.291	0.029
GPR97	-0.102	0.313	-0.054	0.499	-0.406	0.000	-1.460	0.003
HTR6	-0.097	0.314	-0.190	0.097	-0.484	0.000	-1.806	0.000
MC1R	0.098	0.317	0.161	0.317	-0.234	0.003	-0.792	0.870

GPR141	0.097	0.318	-0.044	0.553	-0.314	0.000	-1.219	0.059
MRGPRG	0.095	0.324	0.067	0.791	-0.257	0.000	-1.001	0.383
CHRM4	-0.082	0.327	-0.204	0.184	-0.269	0.007	-1.310	0.001
AGTR1	0.095	0.328	-0.046	0.538	-0.100	0.144	-1.507	0.003
CELSR1	0.096	0.328	0.004	0.822	0.118	0.077	-0.856	0.884
BAI2	-0.103	0.332	0.190	0.226	-0.255	0.000	-1.001	0.390
NTSR2	-0.081	0.335	-0.146	0.420	-0.996	0.000	-1.738	0.000
HCAR1	0.093	0.336	0.122	0.495	0.021	0.737	-0.950	0.536
CCR7	-0.080	0.345	-0.489	0.000	-0.668	0.000	-1.728	0.000
S1PR2	0.078	0.354	-0.042	0.876	-0.698	0.000	-1.492	0.000
CXCR1	-0.089	0.358	-0.166	0.125	-0.406	0.000	-1.297	0.026
GPR37L1	0.076	0.360	0.046	0.334	-0.053	0.559	-0.767	0.732
MRGPRX2	-0.083	0.361	-0.104	0.679	-0.379	0.000	-0.975	0.108
SCTR	-0.079	0.362	-0.201	0.213	-0.409	0.000	-0.686	0.866
TACR3	-0.089	0.363	-0.037	0.586	-0.173	0.009	-1.393	0.008
MAS1	0.088	0.364	0.111	0.540	-0.063	0.370	-0.958	0.527
MRGPRX4	-0.075	0.367	-0.191	0.226	-0.406	0.000	-1.283	0.002
DRD4	-0.087	0.371	-0.420	0.002	-0.474	0.000	-1.660	0.000
GALR1	0.087	0.372	-0.009	0.745	-0.299	0.000	-1.292	0.026
CD97	0.085	0.374	0.010	0.857	-0.009	0.895	-1.082	0.213
GPR156	-0.085	0.375	-0.268	0.076	-0.342	0.000	-1.624	0.000
S1PR1	-0.074	0.376	-0.073	0.896	-0.210	0.030	-0.736	0.884
GPR124	0.084	0.380	0.014	0.881	-0.109	0.101	-0.902	0.695
GPR148	-0.085	0.382	-0.123	0.239	-0.273	0.000	-1.485	0.003
FZD7	0.083	0.388	-0.086	0.358	-0.207	0.007	-1.275	0.033
OPN5	-0.085	0.392	-0.057	0.482	-0.340	0.000	-1.320	0.023
PPYR1	0.082	0.397	0.097	0.615	0.041	0.536	-0.827	0.993
GIPR	0.069	0.406	0.058	0.281	-0.207	0.028	-1.002	0.082
BAI3	0.081	0.406	0.095	0.630	-0.189	0.017	-1.147	0.135
GNAT2	-0.069	0.407	-0.035	0.826	-0.319	0.001	-0.847	0.415
CCR2	0.080	0.407	-0.112	0.279	-0.127	0.079	-1.321	0.016
GPR113	0.070	0.407	0.070	0.237	-0.198	0.038	-1.050	0.047
PTGER1	-0.069	0.409	-0.059	0.997	-0.619	0.000	-1.126	0.013
GPR114	0.079	0.412	0.051	0.889	-0.172	0.013	-1.322	0.019
GPR26	0.079	0.413	0.052	0.887	-0.439	0.000	-1.131	0.160
CXCR4	-0.082	0.414	-0.054	0.502	-0.639	0.000	-1.512	0.002
P2RY2	0.080	0.415	-0.054	0.495	-0.209	0.004	-1.363	0.012
ADRB1	-0.068	0.422	-0.050	0.942	-0.334	0.001	-1.275	0.002
GPR155	-0.078	0.431	0.094	0.630	-0.256	0.000	-1.285	0.030
GPR77	-0.065	0.433	-0.244	0.095	-0.378	0.000	-1.733	0.000
HTR7	-0.074	0.441	-0.215	0.068	-0.438	0.000	-1.254	0.043

P2RY13	-0.074	0.443	-0.067	0.432	-0.285	0.000	-1.242	0.054
PRLHR	-0.064	0.445	-0.172	0.300	-0.381	0.000	-1.067	0.032
HTR3B	0.073	0.451	0.154	0.348	0.053	0.406	-0.662	0.413
EDNRB	0.062	0.456	0.036	0.381	0.072	0.421	-0.641	0.648
CASR	-0.078	0.458	0.112	0.541	-0.210	0.003	-1.060	0.263
P2RY4	0.072	0.458	0.122	0.486	0.016	0.794	-0.885	0.779
CHRM1	-0.071	0.464	-0.243	0.048	-0.489	0.000	-1.700	0.000
ADRB3	-0.060	0.471	-0.064	0.958	-0.119	0.193	-0.991	0.087
GABBR2	0.070	0.472	0.198	0.200	0.096	0.133	-0.779	0.815
HTR1B	-0.073	0.472	0.071	0.767	-0.064	0.374	-0.981	0.441
CCR5	-0.069	0.473	0.065	0.800	-0.278	0.000	-0.995	0.407
HCRTR2	0.068	0.482	-0.099	0.315	-0.170	0.011	-1.446	0.004
HTR1D	0.068	0.485	0.149	0.374	-0.290	0.000	-1.458	0.006
ADRA1A	-0.067	0.489	-0.176	0.113	-0.520	0.000	-1.575	0.001
ELTD1	-0.057	0.489	-0.113	0.611	-0.565	0.000	-1.028	0.064
GPR149	-0.066	0.492	-0.065	0.451	-0.413	0.000	-1.236	0.046
GPRC6A	0.056	0.495	0.003	0.564	-0.534	0.000	-1.276	0.002
GNRHR	0.056	0.495	0.078	0.209	-0.064	0.480	-0.580	0.405
SORCS3	0.066	0.498	0.109	0.552	-0.349	0.000	-1.618	0.001
SSTR1	-0.065	0.501	-0.179	0.111	-0.264	0.000	-1.241	0.043
GPR62	0.056	0.503	0.090	0.170	0.011	0.903	-0.635	0.629
PTGER3	0.064	0.506	0.079	0.716	-0.166	0.011	-1.001	0.386
GPR39	-0.064	0.507	-0.194	0.082	-0.470	0.000	-1.803	0.000
GPR123	0.054	0.515	-0.011	0.660	-0.190	0.039	-1.029	0.055
CNR1	0.062	0.518	0.120	0.501	-0.152	0.029	-0.980	0.454
GPR153	-0.063	0.520	-0.008	0.748	-0.583	0.000	-1.996	0.000
GABBR1	0.053	0.524	-0.010	0.656	-0.120	0.190	-0.937	0.166
OPRK1	0.054	0.524	0.041	0.360	-0.094	0.298	-0.665	0.759
CALCRL	-0.061	0.532	-0.085	0.358	-0.248	0.000	-1.082	0.203
TRHR	0.060	0.536	-0.020	0.689	-0.062	0.340	-0.967	0.479
PTH1R	0.059	0.537	-0.006	0.766	0.035	0.587	-1.082	0.240
GPR125	0.060	0.538	0.126	0.472	-0.081	0.253	-0.910	0.674
LPHN3	0.059	0.538	0.014	0.883	-0.170	0.014	-1.115	0.161
GPR126	-0.062	0.540	-0.012	0.729	-0.012	0.850	-0.878	0.789
GPR146	-0.051	0.540	-0.169	0.314	-0.268	0.005	-1.349	0.000
GPR56	-0.052	0.544	-0.049	0.927	-0.435	0.000	-0.671	0.796
GPR82	0.058	0.551	0.126	0.465	-0.069	0.301	-0.829	0.985
LPAR3	-0.049	0.558	-0.185	0.249	-0.148	0.108	-1.178	0.008
GPR34	-0.056	0.559	-0.013	0.722	-0.273	0.000	-0.902	0.703
OXTR	-0.049	0.566	-0.037	0.840	-0.236	0.011	-0.802	0.572
CRHR2	-0.055	0.575	0.028	0.971	-0.301	0.000	-0.987	0.421

EMR4P	0.046	0.577	-0.127	0.529	-0.013	0.883	-1.502	0.000
GPR108	0.045	0.589	-0.088	0.795	-0.195	0.034	-1.481	0.000
HTR2C	-0.053	0.590	-0.015	0.706	-0.100	0.167	-1.111	0.168
LGR6	-0.051	0.597	-0.198	0.089	-0.268	0.000	-1.305	0.034
PTH2R	0.044	0.599	0.056	0.292	-0.142	0.122	-0.684	0.853
CXCR5	0.044	0.601	-0.089	0.782	-0.357	0.000	-1.355	0.000
GRM1	0.050	0.603	0.128	0.458	-0.083	0.233	-0.946	0.555
GPR179	-0.049	0.610	-0.010	0.736	-0.121	0.079	-1.006	0.380
DRD1	-0.048	0.614	-0.268	0.046	-0.459	0.000	-1.492	0.003
GPR45	-0.049	0.616	-0.339	0.006	-0.199	0.011	-1.354	0.011
GPR75	-0.042	0.617	-0.091	0.773	-0.209	0.026	-0.904	0.222
ADRA2A	-0.041	0.619	-0.209	0.175	-0.267	0.005	-1.317	0.001
OPN4	0.041	0.621	-0.070	0.919	-0.407	0.000	-1.585	0.000
HTR4	-0.041	0.624	-0.109	0.640	-0.300	0.002	-1.293	0.002
GPR133	-0.040	0.625	-0.253	0.080	-0.636	0.000	-1.247	0.002
BDKRB2	0.040	0.628	-0.029	0.782	-0.043	0.640	-0.724	0.945
GHRHR	-0.048	0.632	-0.477	0.000	-0.464	0.000	-1.916	0.000
FPR2	-0.046	0.635	-0.240	0.041	-0.359	0.000	-1.692	0.000
NPY5R	-0.045	0.639	-0.090	0.343	-0.355	0.000	-1.440	0.004
OPN1LW	0.045	0.640	0.116	0.513	-0.353	0.000	-0.928	0.609
MCHR2	-0.045	0.641	-0.184	0.098	-0.216	0.002	-1.555	0.002
GPR143	0.038	0.644	0.021	0.466	-0.209	0.024	-0.870	0.319
F2RL2	-0.039	0.645	-0.010	0.659	-0.423	0.000	-0.963	0.121
FPR3	-0.044	0.645	-0.052	0.512	-0.599	0.000	-1.198	0.065
GPR63	0.044	0.650	0.070	0.774	-0.107	0.110	-0.868	0.829
GRM8	-0.037	0.653	-0.117	0.601	-0.132	0.151	-1.044	0.041
LHCGR	0.037	0.654	0.091	0.169	0.012	0.892	-0.471	0.129
RXFP2	0.042	0.663	0.044	0.932	0.009	0.893	-1.140	0.119
GPR158	0.041	0.666	0.083	0.697	-0.155	0.018	-0.867	0.836
NPY2R	-0.036	0.666	-0.079	0.853	-0.493	0.000	-1.457	0.000
TACR1	0.042	0.667	-0.044	0.552	-0.192	0.007	-1.166	0.091
OXGR1	-0.040	0.677	-0.157	0.161	-0.214	0.001	-1.043	0.275
GPR68	0.040	0.679	-0.201	0.073	-0.204	0.005	-1.301	0.023
FZD6	-0.040	0.683	-0.170	0.130	-0.416	0.000	-1.497	0.002
GPRC5A	0.039	0.687	0.027	0.963	-0.207	0.002	-1.220	0.060
AVPR2	0.038	0.689	0.092	0.644	-0.184	0.008	-0.802	0.906
GPR144	0.038	0.694	0.038	0.969	-0.390	0.000	-1.477	0.003
VN1R2	-0.037	0.696	-0.027	0.641	-0.414	0.000	-1.285	0.027
HCAR2	0.038	0.697	-0.043	0.550	-0.341	0.000	-1.291	0.032
OPRD1	0.038	0.699	0.023	0.940	0.008	0.898	-1.167	0.100
GALR2	0.037	0.701	0.050	0.894	-0.170	0.013	-1.306	0.026

OMG	0.031	0.706	0.081	0.198	-0.110	0.228	-0.915	0.222
GRM6	0.036	0.709	0.082	0.701	-0.098	0.161	-1.059	0.254
NPBWR2	0.036	0.710	0.085	0.684	-0.075	0.262	-1.028	0.314
GPR32	0.030	0.713	-0.031	0.799	-0.151	0.105	-1.074	0.033
PTGER2	-0.035	0.714	0.108	0.556	-0.258	0.000	-1.125	0.152
NMBR	0.035	0.716	0.020	0.915	-0.244	0.000	-1.216	0.066
GPR12	0.035	0.717	0.025	0.947	-0.055	0.395	-1.100	0.180
P2RY1	0.036	0.718	0.027	0.965	-0.188	0.010	-1.063	0.278
HTR2A	0.030	0.719	-0.083	0.825	-0.246	0.015	-1.420	0.000
GPR52	0.035	0.720	-0.074	0.401	-0.107	0.101	-1.322	0.022
S1PR3	0.029	0.721	-0.046	0.905	0.108	0.247	-0.685	0.861
ADRA2C	0.029	0.724	-0.202	0.196	-0.678	0.000	-2.033	0.000
GRM5	0.033	0.730	0.163	0.320	0.080	0.251	-0.590	0.230
BRS3	0.029	0.732	-0.056	0.979	-0.082	0.359	-1.092	0.024
GPBAR1	-0.028	0.737	-0.031	0.797	-0.805	0.000	-1.020	0.061
NMUR2	-0.033	0.740	-0.035	0.602	-0.194	0.008	-1.235	0.048
GPR176	0.027	0.741	-0.099	0.729	-0.054	0.543	-0.834	0.447
GPR171	-0.032	0.744	-0.016	0.701	-0.227	0.001	-0.920	0.645
GPR173	0.031	0.748	0.065	0.803	-0.025	0.696	-1.001	0.387
FZD9	-0.031	0.748	-0.013	0.718	-0.528	0.000	-1.287	0.029
C5AR1	0.031	0.749	0.010	0.859	-0.316	0.000	-1.290	0.029
LPHN1	0.030	0.754	0.058	0.845	-0.106	0.097	-0.779	0.815
P2RY6	0.030	0.756	0.093	0.636	0.002	0.972	-0.634	0.344
GPR1	0.030	0.758	0.176	0.267	-0.293	0.000	-0.659	0.409
LTB4R2	0.029	0.761	0.082	0.702	-0.372	0.000	-1.341	0.016
CCR6	-0.029	0.762	0.144	0.392	-0.380	0.000	-0.977	0.453
EDNRA	0.025	0.762	-0.033	0.814	-0.357	0.001	-1.219	0.003
VN1R5	0.029	0.764	0.176	0.266	-0.204	0.003	-0.853	0.889
CCR9	0.029	0.768	0.034	0.997	-0.312	0.000	-1.008	0.382
NPR1	0.028	0.771	0.134	0.431	-0.022	0.741	-0.835	0.962
GPR162	-0.028	0.771	0.022	0.933	-0.240	0.001	-1.175	0.089
ADRA2B	-0.024	0.773	-0.246	0.087	-0.951	0.000	-1.956	0.000
DRD2	-0.027	0.776	-0.149	0.183	-0.522	0.000	-1.192	0.078
MRGPRE	-0.023	0.781	-0.093	0.750	-0.405	0.000	-1.160	0.013
GPR151	0.027	0.782	-0.011	0.733	0.003	0.962	-1.135	0.140
MAS1L	-0.026	0.782	-0.013	0.721	-0.191	0.012	-0.779	0.821
CCKAR	0.027	0.783	-0.200	0.112	0.006	0.923	-1.339	0.016
FZD8	-0.027	0.785	-0.491	0.000	-0.477	0.000	-1.580	0.001
GPR55	0.022	0.789	0.079	0.212	-0.446	0.000	-0.783	0.660
EMR1	0.026	0.790	0.109	0.558	-0.074	0.301	-1.093	0.190
GPR35	0.022	0.792	-0.086	0.800	-0.021	0.813	-0.959	0.127

FZD4	-0.025	0.794	0.055	0.868	-0.522	0.000	-1.436	0.007
GPR115	0.021	0.796	0.002	0.579	-0.290	0.003	-0.732	0.904
GPR61	-0.021	0.799	-0.129	0.518	-0.317	0.002	-1.530	0.000
CX3CR1	-0.025	0.800	-0.099	0.308	-0.309	0.000	-1.465	0.003
KISS1R	0.021	0.802	0.056	0.300	-0.133	0.144	-1.031	0.058
NPY	0.021	0.803	-0.142	0.452	-0.020	0.825	-1.136	0.012
GPR112	-0.024	0.805	-0.023	0.665	-0.219	0.002	-1.160	0.106
LANCL1	-0.024	0.805	-0.068	0.437	-0.242	0.001	-1.113	0.153
PTAFR	-0.020	0.808	-0.103	0.681	-0.514	0.000	-1.479	0.000
CRY1	-0.024	0.808	0.050	0.898	-0.213	0.003	-0.976	0.457
AVPR1A	-0.020	0.810	-0.087	0.798	-0.311	0.002	-1.156	0.009
FFAR3	-0.024	0.810	0.130	0.446	-0.065	0.332	-0.686	0.487
NTSR1	0.020	0.812	-0.100	0.704	-0.234	0.015	-1.201	0.004
GPR110	-0.020	0.813	0.005	0.555	-0.206	0.028	-0.801	0.594
HCAR3	-0.019	0.814	-0.228	0.124	-0.634	0.000	-1.556	0.000
CCR1	-0.022	0.819	0.136	0.426	-0.116	0.089	-0.807	0.928
PTGER4	0.022	0.824	0.053	0.875	-0.314	0.000	-1.019	0.332
FZD10	-0.021	0.830	-0.038	0.584	-0.574	0.000	-1.325	0.019
P2RY12	0.020	0.835	-0.116	0.266	-0.439	0.000	-1.241	0.064
LEPR	-0.021	0.837	0.082	0.709	-0.251	0.000	-0.926	0.618
CYSLTR1	-0.017	0.837	-0.169	0.321	0.013	0.887	-0.805	0.558
GPR87	0.019	0.842	-0.008	0.745	-0.113	0.095	-1.002	0.398
XCR1	0.019	0.844	0.035	0.990	-0.151	0.024	-1.138	0.136
GPR161	0.019	0.845	0.004	0.822	-0.095	0.139	-1.274	0.032
HTR1F	0.019	0.846	0.005	0.835	-0.316	0.000	-1.281	0.028
VIPR2	0.019	0.848	0.096	0.626	-0.200	0.003	-1.183	0.105
CALCR	0.016	0.848	0.034	0.392	0.010	0.908	-0.694	0.902
CXCR3	0.018	0.849	-0.015	0.705	-0.419	0.000	-1.565	0.001
AVPR1B	0.018	0.852	0.083	0.698	-0.446	0.000	-1.255	0.043
NPBWR1	0.018	0.853	-0.023	0.666	-0.074	0.251	-0.987	0.422
GPR88	0.018	0.855	0.032	0.991	-0.805	0.000	-1.523	0.002
TMEM11	0.017	0.861	-0.053	0.502	-0.228	0.001	-1.179	0.086
GPR160	0.017	0.862	-0.049	0.527	-0.378	0.000	-1.140	0.121
QRFPR	-0.016	0.869	-0.015	0.706	-0.505	0.000	-1.570	0.001
VN1R1	-0.016	0.870	0.183	0.259	0.032	0.630	-0.445	0.061
CXCR2	0.015	0.873	-0.079	0.380	-0.418	0.000	-1.267	0.038
GPR37	-0.013	0.873	-0.070	0.916	-0.833	0.000	-1.770	0.000
FFAR4	0.016	0.874	0.078	0.726	-0.221	0.012	-1.173	0.109
GPR85	0.015	0.875	0.002	0.804	-0.278	0.000	-1.112	0.176
P2RY10	0.015	0.877	0.079	0.725	-0.191	0.004	-0.861	0.865
GRM3	0.013	0.877	-0.088	0.787	-0.321	0.002	-1.087	0.027

GCGR	-0.014	0.883	-0.191	0.088	-0.527	0.000	-1.470	0.004
SUCNR1	0.014	0.884	0.023	0.935	-0.397	0.000	-1.224	0.053
VN1R4	0.012	0.888	-0.056	0.977	-0.168	0.070	-0.791	0.614
GPR42	0.011	0.896	0.011	0.520	-0.192	0.037	-0.687	0.869
RXFP3	0.011	0.899	-0.109	0.642	-0.288	0.005	-1.181	0.008
PTGIR	0.010	0.909	-0.006	0.628	-0.199	0.033	-0.859	0.358
GPR139	-0.011	0.910	-0.298	0.013	-0.374	0.000	-1.863	0.000
MRGPRX1	-0.009	0.925	-0.162	0.136	-0.216	0.003	-1.197	0.069
P2RY8	-0.009	0.925	-0.064	0.454	-0.538	0.000	-1.507	0.002
FZD1	-0.008	0.931	-0.048	0.534	-0.190	0.005	-1.289	0.025
NPR3	-0.008	0.937	0.033	0.999	-0.363	0.000	-1.205	0.065
GRM4	0.007	0.939	0.063	0.819	-0.124	0.066	-0.935	0.575
FFAR2	0.007	0.940	0.091	0.652	-0.408	0.000	-1.306	0.022
NPY1R	0.007	0.944	0.030	0.981	-0.462	0.000	-1.322	0.022
ADORA2B	-0.005	0.956	-0.147	0.166	-0.506	0.000	-1.679	0.000
HRH3	0.005	0.956	-0.007	0.751	-0.282	0.000	-1.060	0.269
GPRC5C	-0.005	0.957	-0.075	0.400	-0.324	0.000	-1.366	0.013
TSHR	0.005	0.962	-0.033	0.617	0.018	0.774	-1.231	0.053
GPR128	-0.004	0.971	0.025	0.952	-0.435	0.000	-1.101	0.185
GPR135	0.003	0.973	0.019	0.915	-0.079	0.238	-0.993	0.414
RXFP1	0.003	0.974	-0.097	0.312	-0.263	0.000	-1.145	0.130
GPR183	-0.003	0.974	0.062	0.819	-0.369	0.000	-1.273	0.033
PTGFR	-0.002	0.980	0.001	0.579	-0.169	0.072	-0.877	0.305
DRD5	0.001	0.988	-0.047	0.534	-0.640	0.000	-1.435	0.007
GPR22	0.000	0.998	-0.075	0.882	-0.108	0.231	-0.939	0.158
ADORA2A	0.000	1.000	0.119	0.502	-0.191	0.007	-0.817	0.966

Supplement table 2. Comparison of the self-renewal efficacy of A549 cells in Figure 3(B). Additive effect is calculated from [# cell in control – (change of # cells in Cisplatin)+(change of cells in siRNA single treatment)].

Treated siRNA		siEMR3	siF2RL3	siGPR108	siNPSR1	siTACR3
Seeding A549 cells		400	400	2,000	800	800
Cell count [96 hr. incubation in fresh media after 96hr. pre-treatment of siRNA and/or Cisplatin]	1) Control	6,436	5,575	7,936	778	7,653
	2) Cisplatin	3,715	3,218	6,265	7,531	6,771
	3) siRNA	4,844	5,707	7,040	4,923	7,908
	Additive effect [2) + 3) - 1)]	2,122	3,350	5,370	4,676	7,027
	**Combination of Cisplatin and siRNA**	**1,246**	**1,967**	**3,721**	**746**	**3,447**

Supplementary Table 3. List of 15 somatic mutations. The survival of patients harboring one or more of these mutations was significantly (p-value <0.01) associated with the RNA expression of the TACR3 gene.

Mutant symbol	Gene name	# Mutants in LUAD	Functions
ABCG4	ATP binding cassette subfamily G member 4	10	**GPCR signaling:**<br>ADGRE2, DOCK11, GUCY2F, RASGRP4, SOS2<br>**Immune response:**<br>ADGRE2, DOCK11, RASGRP4, SELPSOS2<br>**Transport:**<br>ABCG4, ADGRE2, EPG5, CHRNG, PPFIA2, SELP, THADA
ADGRE2	adhesion G protein-coupled receptor E2	10	
CHRNG	cholinergic receptor nicotinic gamma subunit	8	
CPA3	carboxypeptidase A3	9	
DOCK11	dedicator of cytokinesis 11	15	
EPG5	ectopic P-granules autophagy protein 5 homolog	14	
GUCY2F	guanylate cyclase 2F, retinal	17	
PPFIA2	PTPRF interacting protein alpha 2	16	
RASGRP4	RAS guanyl releasing protein 4	8	
SCML2	Scm polycomb group protein like 2	8	
SELP	selectin P	15	
SIM1	SIM bHLH transcription factor 1	13	
SOS2	SOS Ras/Rho guanine nucleotide exchange factor 2	8	
TDRD7	tudor domain containing 7	11	
THADA	THADA armadillo repeat containing	9	
**Total**		**109**	
